# Dynamic evolution of ceftazidime–avibactam resistance due to interchanges between *bla*_KPC-2_ and *bla*_KPC-145_ during treatment of *Klebsiella pneumoniae* infection

**DOI:** 10.3389/fcimb.2023.1244511

**Published:** 2023-08-21

**Authors:** Yili Chen, Runshi Yang, Penghao Guo, Pingjuan Liu, Jiankai Deng, Zhongwen Wu, Qingping Wu, Junqi Huang, Kang Liao

**Affiliations:** ^1^Department of Laboratory Medicine, The First Affiliated Hospital of Sun Yat-sen University, Guangzhou, China; ^2^Guangdong Provincial Key Laboratory of Microbial Safety and Health, Key Laboratory of Agricultural Microbiomics and Precision Application, Ministry of Agriculture and Rural Affairs, State Key Laboratory of Applied Microbiology Southern China, Institute of Microbiology, Guangdong Academy of Sciences, Guangzhou, China; ^3^Organ Transplant Center, The First Affiliated Hospital of Sun Yat-sen University, Guangzhou, China; ^4^Guangdong Provincial Key Laboratory of Organ Donation and Transplant Immunology, Guangzhou, China; ^5^Guangdong Provincial International Cooperation Base of Science and Technology (Organ Transplantation), Guangzhou, China

**Keywords:** carbapenem resistance, *Klebsiella pneumoniae*, ceftazidime-avibactam resistance, KPC-2, KPC-145

## Abstract

**Background:**

The emergence of ceftazidime–avibactam (CZA) resistance among carbapenem-resistant *Klebsiella pneumoniae* (CRKP) is of major concern due to limited therapeutic options.

**Methods:**

In this study, 10 CRKP strains were isolated from different samples of a patient with CRKP infection receiving CZA treatment. Whole-genome sequencing (WGS) and conjugation experiments were performed to determine the transferability of the carbapenem resistance gene.

**Results:**

This infection began with a KPC-2-producing *K. pneumoniae* (CZA MIC = 2 μg/mL, imipenem MIC ≥ 16 μg/mL). After 20 days of CZA treatment, the strains switched to the amino acid substitution of T263A caused by a novel KPC*-*producing gene, *bla*_KPC-145_, which restored carbapenem susceptibility but showed CZA resistance (CZA MIC ≥ 256 μg/mL, imipenem MIC = 1 μg/mL). The *bla*_KPC-145_ gene was located on a 148,185-bp untransformable IncFII-type plasmid. The subsequent use of carbapenem against KPC-145-producing *K. pneumoniae* infection led to a reversion of KPC-2 production (CZA MIC = 2 μg/mL, imipenem MIC ≥ 16 μg/mL). WGS analysis showed that all isolates belonged to ST11-KL47, and the number of SNPs was 14. This implied that these *bla*_KPC_-positive *K. pneumoniae* isolates might originate from a single clone and have been colonized for a long time during the 120-day treatment period.

**Conclusion:**

This is the first report of CZA resistance caused by *bla*_KPC-145_, which emerged during the treatment with CZA against *bla*_KPC-2_-positive *K. pneumoniae*-associated infection in China. These findings indicated that routine testing for antibiotic susceptibility and carbapenemase genotype is essential during CZA treatment.

## Introduction

With the extensive application of carbapenems, carbapenem-resistant *Enterobacterales* (CRE) infections have been a major threat to public healthcare, especially carbapenem-resistant *Klebsiella pneumoniae* (CRKP)-associated infections (Kim et al., 2017; Plazak et al., 2018). The China Antimicrobial Surveillance Network (CHINET) reported that there is an upward trend in carbapenem resistance in *Klebsiella pneumoniae* (Hu et al., 2019). CRE-related infections caused the deaths of 42.1% of patients with infections and 73% of severely infected patients who were in septic shock (Daikos et al., 2014; Xu et al., 2017). The production of *Klebsiella pneumoniae* carbapenemase (KPC), especially KPC-2, is dominant among CRKP ST11 strains in China (Yang et al., 2013; Zhang et al., 2017).

Ceftazidime–avibactam (CZA) has been approved as an effective antibiotic for CRE treatment. However, the emergency of CZA resistance rapidly appeared after its widespread application for the treatment of CRE infections (Shields et al., 2016). Some sporadic reports of CZA resistance were observed after the use of CZA for the treatment of KPC in China, such as in Shanghai (Shi et al., 2020) and Henan (Li et al., 2021a). Nevertheless, there are currently no reports of CZA developing resistance after use in southern China. In this study, we reported the dynamic evolution of CZA resistance due to a mutation between plasmid-borne *bla*_KPC-2_ and *bla*_KPC-145_ by sequencing clinical isolates from a patient with CRKP infection undergoing CZA treatment. To our knowledge, this is the first report on KPC-145 producing CRKP, which harbored a new amino acid substitution site on the basis of KPC-33.

## Patients and methods

### Sample source and identification

Ten strains of *K. pneumoniae* were isolated from a 32-year-old man who was diagnosed with severe acute pancreatitis and multiple organ failure on admission to the First Affiliated Hospital of Sun Yat-sen University, Guangzhou. Empiric treatment with tigecycline, imipenem, and caspofungin was adopted, and surgical drainage was performed gradually from around the liver and around the pancreas. The patient developed septic shock 20 days after admission. The first *K. pneumoniae* strain (CZHKP-01) was obtained from a blood culture before CZA treatment. The third strain (CZHKP-03) was isolated from stool specimens. The remaining eight strains were isolated from the drainage fluid of the abdominal cavity. Species identification was performed by matrix-assisted laser desorption ionization–time-of-flight mass spectrometry (MALDI-TOF MS) (bioMérieux, Marcy l’Etoile, France).

### Antimicrobial susceptibility testing

Antimicrobial susceptibilities for the strains were detected by Gram-negative susceptibility (GNS) cards on the Vitek-2 system (bioMérieux, Marcy l’Etoile, France). MICs of tigecycline and CZA were determined by broth microdilution method. Susceptibility testing results were interpreted under the criteria recommended by the Clinical and Laboratory Standards Institute (CLSI, 2022). The quality control strain for susceptibility testing was *Escherichia coli* ATCC 25922 and *K. pneumoniae* ATCC 700603.

### Carbapenemase phenotype and genotype detection

The modified carbapenem inactivation method (mCIM) was carried out according to the recommendations of the CLSI (M100-S29). A carbapenemase inhibitor enhancement testing kit was purchased from Zhuhai Deere Bioengineering Co. Ltd. (Zhuhai, Guangdong, China), and the assay was performed as previously described (Tsakris et al., 2010).

Xpert Carba-R (Cepheid, Sunnyvale, CA, USA), NG-Test^®^ CARBA 5 (NG Biotech, Guipry, France), and Goldstream Carbapenem-resistant K.N.I.V.O. Detection K-Set (Beijing Gold Mountain River Tech Development Co. Ltd., Beijing, China) were applied for rapid detection of 10 strains carbapenemase genotype according to the instructions. Targeted PCR was performed. The primers KPC1F (5′-GCTACACCTAGCTCCACCTTC-3′) and KPC1R (5′-GCATGGATTACCAACCACTGT-3′) were used to sequence the entire open reading frame (ORF) of the *bla*_KPC_ gene (Smith Moland et al., 2003).

### Transferability of carbapenem resistance gene

The transferability of the carbapenem resistance phenotype was determined by conjugation experiments (Lorenzo-Diaz and Espinosa, 2009) using the streptomycin-resistant *E. coli* C600 strain. Transconjugants were chosen on MacConkey agar plates (Huankai Co. Ltd., Guangzhou, China) supplemented with cefotaxime (1 mg/L) and streptomycin (1,500 mg/L).

The electrotransformation experiments (Choi et al., 2006) were further performed for isolates that failed conjugation experiments. In brief, plasmid DNA was extracted using a QIAPrep Plasmid Midi Kit (QIAGEN, Hilden, Germany) and transformed into electro-competent *E. coli* DH5α (TaKaRa Biotechnology, Dalian, China). electrotransformants were selected on LB agar plates (Huankai Co. Ltd., Guangzhou, China) supplemented with cefotaxime (1 mg/L).

We further confirmed the transconjugants or electrotransformants by PCR for the *bla*_KPC-2_ gene and tested for antimicrobial susceptibility as described above.

### Cloning of mutant *bla*_KPC-145_ gene

Plasmid DNA was extracted from parent strains, and primers targeting the entire ORF of the *bla*_KPC-2_ gene and *bla*_KPC-145_ gene, along with *Nco*I and *Bam*HI restriction sites, were designed and used for PCR. The PCR products and the plasmid vector pET28a were digested with the restriction enzymes *Nco*I*–Bam*HI (TaKaRa Biotechnology, Dalian, China) and then ligated at 16°C overnight. The recombinant plasmids were transformed into *E. coli* BL21 (DE3); all the transformants were confirmed by PCR and sequencing analysis. Sodium dodecyl sulfate-polyacrylamide gel electrophoresis (SDS-PAGE) was conducted to verify the expression of the target proteins KPC-2 and KPC-145. Antimicrobial susceptibility testing for the BL21 transformants was performed as mentioned above.

### Whole-genome sequencing and bioinformatics analysis

Genomic DNA of the selected isolates was extracted using a DNA extraction kit (Magen, Guangzhou, China), and 150 bp paired-end reads were obtained using an Illumina NextSeq 550 system (Illumina, San Diego, CA, USA). For each whole-genome sequencing (WGS) data, at least 100-fold coverage of original reads was obtained, and the assembly draft of the sequences was generated by SPAdes (Bankevich et al., 2012). Furthermore, a representative strain (CZHKP-07) was selected for further sequencing by using the MinION platform (Oxford Nanopore Technologies, Oxford, UK).

A hybrid assembly of both short Illumina NextSeq reads and long MinION reads was built using Unicycler with the Pilon option to modify the assembled readings (Walker et al., 2014; Wick et al., 2017) and annotated with RAST (Aziz et al., 2008) and Prokka (Seemann, 2014) for functionality. The PCR-based replicon typing (PBRT) and replicon sequencing typing (RST) methods were applied to characterize IncF plasmids, as described previously (Carattoli et al., 2005; Villa et al., 2010). Prophage analysis was conducted by PHAST (http://phaster.ca/). Multilocus sequence types (MLST) and antibiotic resistance genes (ARGs) were analyzed using ABRicate (https://github.com/tseemann/abricate). Capsule serotype and virulence genes (yersiniabactin, colibactin, and other siderophore locus) were determined using Kleborate (Lam et al., 2021). Core genome alignment and calling of single nucleotide polymorphisms (SNPs) were carried out using the Snippy (https://github.com/tseemann/snippy) against reference strain CZHKP-07 and followed by purging using Gubbins (Croucher et al., 2015). The heatmap showing the SNP matrix was generated by using the “pheatmap” package in RStudio; core-genome SNPs were used to construct a maximum-likelihood phylogenetic tree by RAxML (Stamatakis, 2014) and visualized by iTOL (Letunic and Bork, 2016).

### Data availability

The sequence of *bla*_KPC-145_ has been deposited in GenBank under Accession No. OP626310.

## Results

### Alterations in drug resistance during treatment

In our case, after initial combination treatment with imipenem (2.0 g, IV, Q8h) and tigecycline (first dose of 100 mg, then 50 mg, IV DRIP, Q12h) for 20 days, *bla*_KPC-2_-positive *K. pneumoniae* strains (CZHKP-01 and CZHKP-02) were isolated from the blood culture and drainage fluid, showing resistance to imipenem and meropenem (**Table 1**). Subsequently, antibiotic therapy was adjusted to combination with CZA (2.5 g, IV DRIP, Q8h), polymyxin B (first dose of 1,000,000 U, then 500,000 U, IV, Q12h), tigecycline, and caspofungin, along with adequate surgical drainage (from perihepatic, lesser omental sac, right peripancreatic, and cystic duct). However, after using CZA for 20 days, the patient’s body temperature rose again. The first CZA resistance *K. pneumoniae* strain CZHKP-03 was isolated from fecal culture followed by CZHKP-05~CZHKP-09 (all isolated from abdominal drainage fluid). These strains restored carbapenem susceptibility but showed CZA resistance (CZA MIC ≥ 256 μg/mL, imipenem MIC = 1 μg/mL). Considering that long-term use of CZA may lead to drug-resistant strains, we stopped using CZA and switched to polymyxin B and tigecycline for anti-infection treatment.

**Table 1:** *Klebsiella pneumoniae* drug susceptibility testing and carbapenemase testing results.

Strain	MIC (μg/mL)					mCIM	Carbapenemase inhibitor enhancement	NG-test CARBA 5	K.N.I.V.O. Detection K-Set	GeneXpert
	Ceftazidime–avibactam	Imipenem	Meropenem	Polymyxin B	Tigecycline					
CZHKP-01	2	≥16	≥16	≤0.5	2	Pos^a^	Pos^a^	KPC	KPC	KPC
CZHKP-02	2	≥16	≥16	≤0.5	2	Pos^a^	Pos^a^	KPC	KPC	KPC
CZHKP-03	≥256	1	4	≤0.5	2	Neg	Neg	Neg	KPC	KPC
CZHKP-04	2	≥16	≥16	≤0.5	2	Pos^a^	Pos^a^	KPC	KPC	KPC
CZHKP-05	≥256	1	4	≤0.5	2	Neg	Neg	Neg	KPC	KPC
CZHKP-06	≥256	1	4	≤0.5	2	Neg	Neg	Neg	KPC	KPC
CZHKP-07	≥256	1	4	≤0.5	2	Neg	Neg	Neg	KPC	KPC
CZHKP-08	≥256	1	4	≤0.5	2	Neg	Neg	Neg	KPC	KPC
CZHKP-09	≥256	1	4	≤0.5	2	Neg	Neg	Neg	KPC	KPC
CZHKP-10	2	≥16	≥16	≤0.5	2	Pos^a^	Pos^a^	KPC	KPC	KPC

a Positive for Ambler class A carbapenemase.

**Figure 1:**
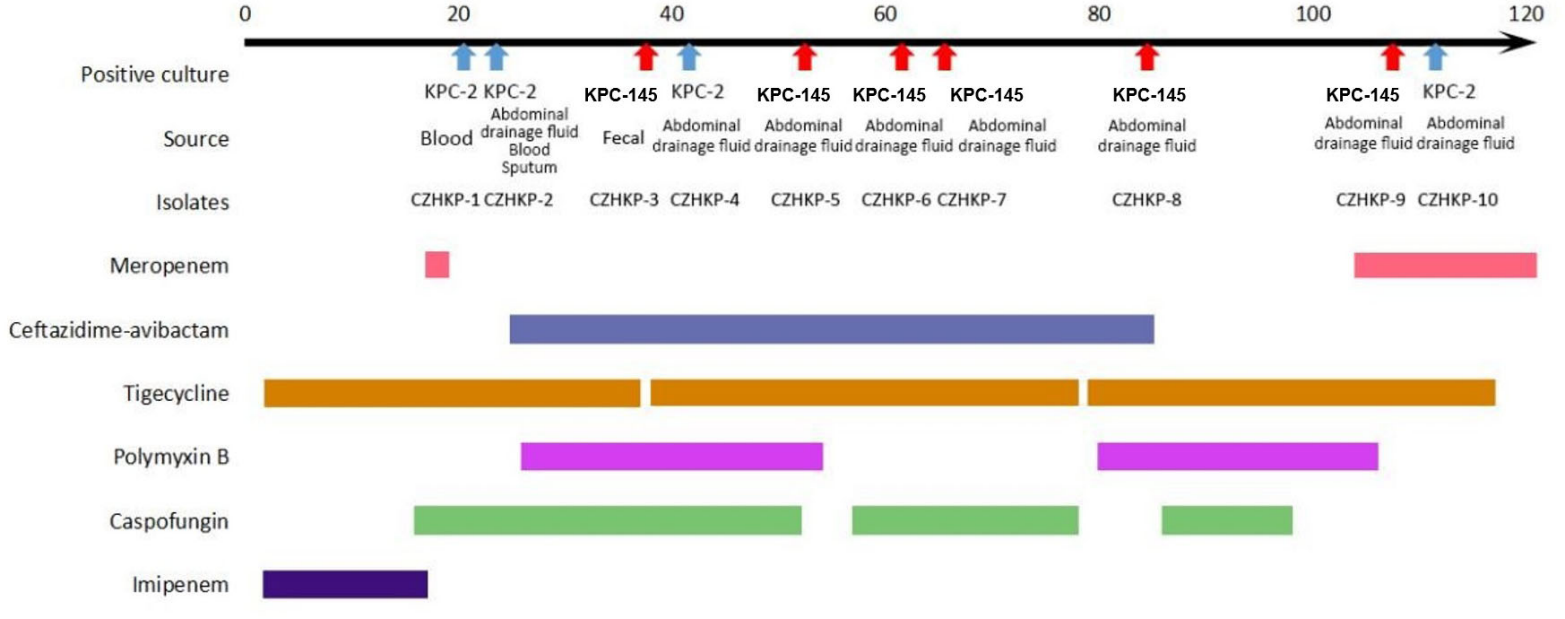
History of the *K. pneumoniae* isolations and the clinical antimicrobial treatment course of a patient with KPC-Kp infection.

On the 105th day of hospitalization, the anti-infection therapy was adjusted to tigecycline combined with meropenem. After over a week of treatment, the heat peak of the patient’s body temperature decreased and the drainage fluid decreased gradually. On the 113th day of hospitalization, CZHKP-10 isolated from abdominal drainage fluid culture recovered susceptibility to CZA (CZA MIC = 2 μg/mL, imipenem MIC ≥ 16 μg/mL). Through sufficient surgical drainage and reasonable anti-infection treatment, the patient’s condition ultimately improved and returned to the regular ward for further treatment on the 120th day of hospitalization. Clinical and microbiological details, timelines, and antibiotic therapies used are summarized in **Figure 1**.

### Detection of carbapenemase

The performance comparison of different carbapenem enzyme detection methods among the 10 strains is shown in **Table 1**. Sanger sequencing results of the *bla*_KPC_ gene revealed that four CZA-susceptible isolates (CZHKP-01, CZHKP-02, CZHKP-04, CZHKP-10) carried the *bla*_KPC-2_ gene. Interestingly, the rest of the six CZA-resistant isolates (CZHKP-03, CZHKP-05, CZHKP-06, CZHKP-07, CZHKP-08, and CZHKP-09) harbored a novel *bla*_KPC_ gene with a point mutation (A to G) at nucleotide position 787 compared with the *bla*_KPC-33_ gene; this mutation resulted in the amino acid substitution of threonine to alanine (T263A), assigned by GenBank as *bla*_KPC-145_.

In addition, the results of mCIM, carbapenemase inhibitor enhancement testing, and NG-Test CARBA 5 showed negative results for *bla_KPC-145_*-positive strains, which indicated that routine clinical detections have some defects on *bla*_KPC-145_-positive strains. Another immunochromatographic method, Goldstream Carbapenem-resistant K.N.I.V.O. Detection K-Set, which showed positive results in detecting *bla*_KPC-145_ strains, seems to have a better cost-to-benefit ratio. Moreover, molecular screening of GeneXpert for *bla*_KPC-145_ showed good sensitivity.

### Transferability *bla*_KPC-2_ and *bla*_KPC-145_ gene

Unfortunately, despite numerous attempts conducted by conjugation assays, the transfer of *bla*_KPC-2_ and *bla*_KPC-145_ genes failed in all 10 K*. pneumoniae* isolates. Electrotransformations were further performed, and 10 electrotransformants were successfully obtained. All of the electrotransformants exhibited resistance to all β-lactams, aminoglycosides, and fosfomycin.

### Cloning of *bla*_KPC-145_ gene

The recombinant plasmid carrying the *bla*_KPC-145_ gene in *E. coli* BL21, which conferred resistance to ceftazidime/avibactam (MIC = 2 mg/L) and displayed decreased carbapenem MICs (imipenem MIC = 0.125 mg/L and meropenem MIC = 0.25 mg/L) compared with *bla*_KPC-2_ in BL21. Moreover, the MICs of cefotaxime, ceftazidime, and aztreonam were 32-, 16-, and 16-fold higher than the original recipient *E. coli* BL21. However, compared with *bla*_KPC-2_, the MICs of *bla*_KPC-145_-carrying electrotransformants to carbapenems were 32- to 128-fold lower than those of *bla*_KPC-2_-carrying transformants.

### WGS analysis and phylogenetic characteristics

A total of 10 KPC-producing strains were subjected to WGS. The *in silico* analysis showed that these 10 strains belonged to ST11-KL47 (associated with the *wzi* allele 209) and harbored 11 resistance genes, conferring resistance to aminoglycoside (*aac3-IId*, *rmtB*), fosfomycin (*fosA3*), quinolone (*qnrS1*), tetracycline (*tetA*), β-lactam (*bla*_KPC-2_ or *bla*_KPC-145_, *bla*_CTX-M-64_, *bla*_TEM-1B_, *bla*_SHV-12_), and trimethoprim/sulfamethoxazole (*sulI*, *dfrA1*). Furthermore, the representative *K. pneumoniae* strain (CZHKP-07) was sequenced using the MinION platform, followed by hybrid assembly. The CZHKP-07 chromosome is 5,448,939 bp in length, with a GC content of 57.44%, and harbors five plasmids with sizes of 230,198 bp (IncFIB and IncFII), 148,185 bp (IncFII), 55,161 bp, 11,970 bp (ColRNAI) and 5,596 bp (ColRNAI), named pCZHKP07-1 to pCZHKP07-5 (**Supplementary Table S1**). The pCZHKP07-2 was an F33:A−:B− plasmid and contained six resistance genes, including *bla*_KPC-145_. Meanwhile, the plasmid pCZHKP07-2 possessed addiction systems (*pemKI*, *hok-sok-mok*, *ccdAB*); these genes promote plasmid maintenance during vertical transmission.

**Figure 2:**
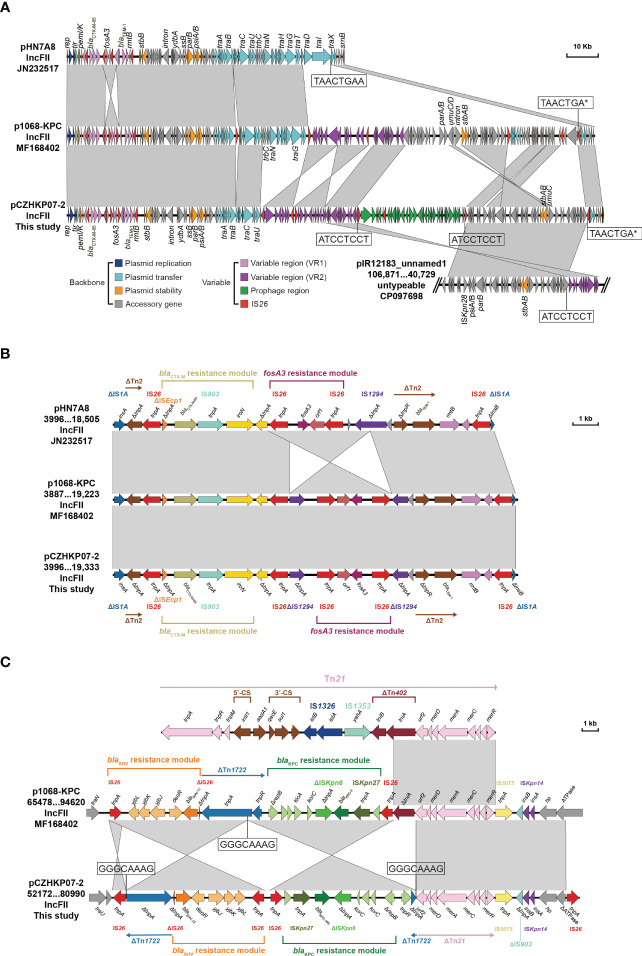
Linear comparison of the KPC-145-positive plasmid pCZHKP07-2 identified in this study with plasmid pHN7A8, p1068-KPC, and pIR12183_unnamed1 **(A)** and two variable regions **(B, C)**. The arrows indicate the positions and transcriptional directions of the ORFs, while homologous regions are represented in gray.

**Figure 3:**
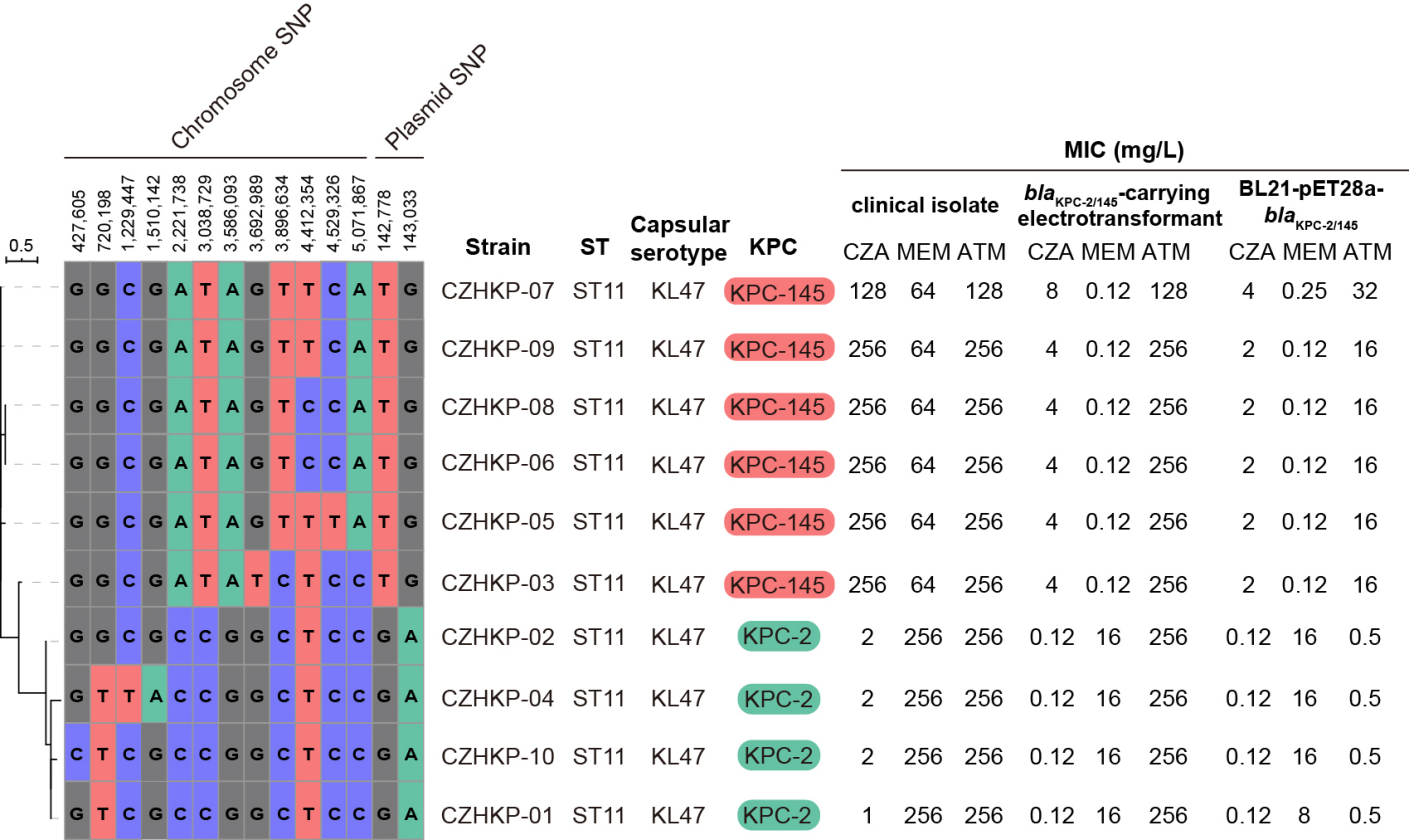
The maximum likelihood phylogenetic tree generated from the core-genome sequences of the 10 KPC-positive strains identified in this study. Each isolate is listed with its ST, capsular serotype, *bla*_KPC_ allele, and antimicrobial susceptibility results.

A linear sequence comparison of the plasmids pCZHKP07-2, pHN7A8 (Accession No. JN232517), and p1068-KPC (Accession No. MF168402) was performed. The genetic structure suggested that pCZHKP07-2 possessed a similar backbone structure as F33:A−:B− plasmid pHN7A8, but a large segment of the conjugative transfer-associated genes (*tra* and *trb*) was absent (**Figure 2A**), which might be the reason for conjugation failure. Therefore, we speculate that the insertion of an extra copy of IS*26* at target site TAACTGAA on the *traI* gene in the pHN7N8 plasmid, followed by homologous recombination between IS*26* elements, may have led to the loss of the conjugative transfer regions. Meanwhile, two variable regions and a prophage region were embedded in the pCZHKP07-2 (**Figure 2A**). The first variable region (VR1) was 15,338 bp in length and was flanked at both ends by fragments of IS*1* found in pCZHKP07-2, which consists of four different resistance genes, *bla*_CTX-M-65_, *fosA3*, *bla*_TEM-1B_, and *rmtB*, and abundant complete or truncated transposons. This structure was similar to the pHN7A8 except for the *fosA3* resistance module with an opposite orientation, which was flanked by two intact copies of IS*26* at both ends (**Figure 2B**). The second variable region (VR2) possessed *bla*_KPC-145_ and *bla*_SHV_ modules. The *bla*_KPC-145_ and *bla*_SHV_ regions in pCZHKP07-2 were disrupted into two separate parts, which may be caused by homologous recombination after IS*26* insertion. The genetic structure of *bla*_KPC-145_ was composed of Tn1722-based unit transposon including IS*Kpn27*-*bla*_KPC-145_-IS*Kpn6*-*korC*-*klcA*-Δ*repB*, and the *bla*_SHV_ module was flanked by two intact or truncated IS*26*, resulting the differed from p1068-KPC with the inversion of this segment (**Figure 2C**). Moreover, the prophage region (30,305 bp) of pCZHKP07-2, flanked by 8 bp ATCCTCCT direct repeats (DRs), was inserted into the plasmid pIR12183_unnamed1 ATPase gene and bound at both ends by IS*26*. A maximum likelihood phylogenetic tree and SNP matrix were constructed based on core-genome SNPs (cgSNPs) from the 10 genomes in this study. We identified four KPC-2-positive *K. pneumoniae* and six KPC-145-positive *K. pneumoniae* that were separated into two clades and shared 14 SNPs in total (**Figure 3**).

## Discussion

KPC-2-producing strains, especially those that belong to ST11, are the majority population of *K. pneumoniae* resistant to carbapenems and become one of the most urgent public health issues in China (Li et al., 2021b). Avibactam is a β-lactamase inhibitor with a wide range of therapeutic effects against serine β-lactamases, including KPC. Combined with ceftazidime, CZA has become an important therapeutic option for treating KPC-producing *K. pneumoniae* infections (Tumbarello et al., 2021). Unfortunately, the development of CZA resistance has increased since it was clinically approved. In the International Network for Optimal Resistance Monitor (INFORM) surveillance program (2015–2017), the susceptibility rate of CZA against CRE was 73.0% (Spiliopoulou et al., 2020). A recent study from the CHINET revealed that CZA exhibited potent activity against *Enterobacterales* (93.6% susceptible), while only 65.2% of CRE remained susceptible to CZA (Guo et al., 2022).

In the present study, the first CZA resistance strain was isolated after treatment with CZA for 20 days, consistent with the previous reports that CZA resistance commonly appears after 10 to 19 days and sometimes after 33 days (Shi et al., 2020). Our study also showed that the *bla*_KPC-145_ gene-mediated CZA resistance was accompanied by a substantial decrease in carbapenem MICs. Moreover, when the therapy was subsequently adjusted to meropenem, it took only 4 days for the strains to change from *bla*_KPC-145_ back to *bla*_KPC-2_. It was observed that the *in vivo* evolution of wild-type KPC-2 changed into KPC-145 and then reversed to its original wild-type KPC-2. Alternatively, the combination of CZA and carbapenems may be an ideal option, which has been proven to be effective in some clinical cases (Bianco et al., 2020; Shi et al., 2020; Li et al., 2021a). In addition, meropenem–vaboractam has been receiving increasing attention as a potential antibiotic for clinics, and there are no *K. pneumoniae*-harboring mutant *bla*_KPC_ resistant to meropenem–vaborbactam (Wilson et al., 2019).

So far, there are three principal CZA resistance mechanisms (Galani et al., 2021), including the production of metallo-β-lactamases, increased expression of KPC carbapenemases with porin mutations, and amino acid changes of KPC carbapenemases. Among them, the primary CZA resistance mechanism is a single mutation within the *bla*_KPC_ gene allele, which is commonly observed in several pivotal regions, especially the omega loop (amino acid positions 164–179), the 240-loop (Galani et al., 2021) (aa 238–243) and 270-loop (aa 263–277) (Venditti et al., 2021). The novel variant, KPC-145, with two amino acid substitutions (D179Y and T263A), was identified in our study. These two amino acid substitutions were observed in KPC-3, named KPC-70 (Carattoli et al., 2021). Our results revealed that KPC-145 mediated the CZA resistance and restored susceptibility to carbapenems, similar to KPC-70 in the previous report. The D179Y mutation within the omega loop may be the main mechanism for resistance change for the KPC-145 enzyme, which causes enhanced affinity to ceftazidime and restricts avibactam binding (Winkler et al., 2015). In addition, it is noteworthy that the number of KPC variants has been increasing rapidly in recent years; this situation poses a significant threat to public health.

Therefore, rapid and accurate detection of KPC-subtype-producing strains plays an important role in clinical drug selection and patient prognosis. However, in our study, the results of mCIM, carbapenemase inhibitor enhancement testing, and NG-Test^®^ CARBA 5 showed negative results for *bla*_KPC-145_-positive strains, which indicated deficiencies in routine clinical testing for *bla*_KPC-145_-positive strains. Both K.N.I.V.O. Detection K-Set and GeneXpert showed satisfactory diagnostic performance in detecting *bla*_KPC-145_, which was consistent with other studies on the detection ability of KPC-2 variants (Ding et al., 2021).

The dissemination of resistance genes is also associated with sequence types and plasmid replicon types. Since the emergence of KPC-producing *K. pneumoniae* in 2001 (Yigit et al., 2001), ST11 has become the most prevalent CRKP clone in Asia, especially in China. In this study, we found that all isolates belonged to ST11-KL47, and the number of SNPs was 14. This implies that these *bla*_KPC_-positive *K. pneumoniae* isolates might originate from a single clone and long-term colonization in this patient during the 120-day therapy. Meanwhile, CZA resistance encoded by *bla*_KPC_ variants has been increasingly reported in ST11 *K. pneumoniae* in China, including *bla*_KPC-33_ (Shi et al., 2020), *bla*_KPC-51_, *bla*_KPC-52_ (Sun et al., 2020), *bla*_KPC-74_ (Li et al., 2021b), and *bla*_KPC-93_ (Wu et al., 2022). Otherwise, ST11 is a single-locus variant of the globally popular high-risk clone ST258 through the acquisition of the *tonB* allele (Breurec et al., 2013). These two sequence types together with ST512 and a few other SNP variants comprising the clonal group CG258, responsible for the global dissemination of CRKP. This underscores the importance of coordinated international action to surveillance and control the high-risk clone type. IncF is a narrow host plasmid that is widely found in *Enterobacteriaceae*. Numerous studies have shown that the IncF subtype IncFII plasmid carries a variety of ARGs and plays a significant role in the transmission of specific resistance genes, including the *bla*_KPC_ gene (Shi et al., 2018). In our study, the untransferable IncFII plasmid carried by *K. pneumoniae* encodes the resistance genes *bla*_KPC-145_, *bla*_TEM-1B_, *bla*_SHV-12_, *bla*_CTX-M-64_, *fosA3*, and *rmtB*, which are associated with CZA, cephalosporins, fosfomycin, and aminoglycosides, suggesting that IncFII plasmid contribute to the transmission of resistant strains and ARGs. The mobile genetic elements were widely distributed in the bacterial genomes and played a crucial role in the dissemination of antibiotic resistance (Partridge et al., 2018). The diverse structure of variable regions may be caused by a series of molecular module rearrangements, acquisitions, or loss events mediated by insertion sequences such as IS*26* by transposition or homologous recombination. It is also worth pointing out that IS*26* not only mediated the inversion of the resistance modules but also caused the absence of large segments of the conjugative transfer region.

## Conclusion

In this study, we reported the first case of KPC-145-producing ST11-KL47 *K. pneumoniae* strains conferring resistance to CZA after exposure to CZA for 20 days. This novel variant mediated CZA resistance and restored susceptibility to carbapenems, indicating that the detection and identification of mutant *bla*_KPC_ isolates may be a key to antimicrobial management.

## Data availability statement

The datasets presented in this study can be found in online repositories. The names of the repository/repositories and accession number(s) can be found in the article/**Supplementary Material**.

## Author contributions

YC, RY, QW, JH, and KL conceived and designed the experiments. YC, JD, and ZW collected clinical data. YC, RY, and PG performed laboratory work. RY performed whole-genome sequencing and bioinformatics analysis. YC, RY, and PL analyzed clinical and microbiological data. YC, RY, and PC prepared the manuscript. All authors contributed to the article and approved the submitted version.
