# Supplementary material for: Dynamic evolution of ceftazidime–avibactam resistance due to interchanges between blaKPC-2 and blaKPC-145 during treatment of Klebsiella pneumoniae infection
Source: Front Cell Infect Microbiol. 2023 Aug 21;13:1244511. doi: 10.3389/fcimb.2023.1244511 (PMC10476102; doi:10.3389/fcimb.2023.1244511)
Supplement: Supplementary file 1 [file Table_1.docx]

Supplementary Table 1. Characteristics of CZHKP-07 chromosome and plasmid sequence information.

| **CZHKP-07** | **Size (bp)** | **ST (chr) / Inc (pls)** | **GC content (%)** | **ARGs** | **Virulence genes** |
| --- | --- | --- | --- | --- | --- |
| chromosome | 5,448,939 | ST11 (3-3-1-1-1-1-4) | 57.44 | *bla*_SHV-187_, *oqxAB*, *fosA* | *mrkABCDFJIH*, *fyuA* , *irp1*, *irp2*, *ybtAEPQSTUX* |
| pCZHKP07-1 | 230,198 | IncFIB, IncFII | 51.44 | *aac3-IId*, *qnrS1*, *sul1*, *dfrA1*, *tetA* | NA |
| pCZHKP07-2 | 148,185 | IncFII | 54.26 | *bla*_KPC-145_, *bla*_CTX-M-64_, *bla*_TEM-1B_, *bla*_SHV-12_, *fosA3*, *rmtB* | NA |
| pCZHKP07-3 | 55,161 | Non-typable | 52.65 | NA | NA |
| pCZHKP07-4 | 11,970 | ColRNAI | 55.58 | NA | NA |
| pCZHKP07-5 | 5,596 | ColRNAI | 51.14 | NA | NA |
